# Understanding the structure and functioning of polar pelagic ecosystems to predict the impacts of change

**DOI:** 10.1098/rspb.2016.1646

**Published:** 2016-12-14

**Authors:** E. J. Murphy, R. D. Cavanagh, K. F. Drinkwater, S. M. Grant, J. J. Heymans, E. E. Hofmann, G. L. Hunt, N. M. Johnston

**Affiliations:** 1 British Antarctic Survey, NERC, Cambridge, UK; 2 Institute of Marine Research, Bergen, Norway; 3 Scottish Association for Marine Science, Oban, Argyll, UK; 4 Center for Coastal and Physical Oceanography, Old Dominion University, Norfolk, VA, USA; 5 School of Aquatic and Fishery Sciences, University of Washington, Seattle, WA, USA

**Keywords:** polar, ocean, ecosystems, climate change, biodiversity, ecosystem functioning

## Abstract

The determinants of the structure, functioning and resilience of pelagic ecosystems across most of the polar regions are not well known. Improved understanding is essential for assessing the value of biodiversity and predicting the effects of change (including in biodiversity) on these ecosystems and the services they maintain. Here we focus on the trophic interactions that underpin ecosystem structure, developing comparative analyses of how polar pelagic food webs vary in relation to the environment. We highlight that there is not a singular, generic Arctic or Antarctic pelagic food web, and, although there are characteristic pathways of energy flow dominated by a small number of species, alternative routes are important for maintaining energy transfer and resilience. These more complex routes cannot, however, provide the same rate of energy flow to highest trophic-level species. Food-web structure may be similar in different regions, but the individual species that dominate mid-trophic levels vary across polar regions. The characteristics (traits) of these species are also different and these differences influence a range of food-web processes. Low functional redundancy at key trophic levels makes these ecosystems particularly sensitive to change. To develop models for projecting responses of polar ecosystems to future environmental change, we propose a conceptual framework that links the life histories of pelagic species and the structure of polar food webs.

## 1. Introduction

Global loss of biodiversity has focused attention on the influence of species composition on ecosystem structure and functioning and the provision of ecosystem services [1]. Studies of terrestrial and marine systems have provided important understanding of the links between biodiversity and ecosystem functioning [2–4]. Most of the marine studies have, however, focused on more static systems (e.g. benthic systems or coral reefs), while understanding of the processes that connect species composition and ecosystem functioning in pelagic and open-ocean ecosystems is generally lacking [5], including in the polar regions.

Rapid changes in multiple climate and oceanic processes are occurring in the Arctic and Antarctic that are affecting ocean circulation, biogeochemistry (including acidification) and sea-ice distribution [6–8]. In addition, direct anthropogenic threats (e.g. fisheries, commerce and pollution) are increasing and both polar ecosystems have experienced extensive and long-term harvesting that has generated top-down changes in food webs [9,10]. Evidence of the ecological impacts of change at different scales is clear in both polar ecosystems [6,7,11–13], and is manifest in changing productivity, population sizes, biological diversity and food-web structure, but the underlying mechanisms involved are unclear.

These ecological changes are influencing overall ecosystem structure and functioning, and the maintenance of services that include: roles in regulating climate processes, supporting fisheries, tourism, local communities (Arctic), and maintaining unique biological diversity [14]. Consideration of the value of polar ocean ecosystem services, and the biodiversity supporting them, has a useful role in the development of sustainable management strategies for these increasingly threatened ecosystems [15]. Understanding the factors determining structure and functioning is fundamental to valuing these ecosystems, and crucial for analyses of the impact of future change on the services they provide [15].

The traditional view of polar ocean food webs, based largely on qualitative analyses, is of short food chains usually represented as a single aggregated network. Although a range of detailed studies have been undertaken [9,13,16–18], these have not been synthesized to provide a broader understanding of how food webs vary across polar habitats. Polar ocean ecosystems are characterized by relatively low metazoan diversity and the apparent dominance of a small number of species in the energy flow between lower and higher trophic levels [9,13,16,17]. In such systems, there is a skew in functional roles, with a small number of species performing most of the core ecological functions (low functional redundancy) [1–3,19–22]. However, the roles of individual species in the processes influencing the structure, functioning and resilience of these ecosystems are poorly understood. Polar pelagic ecosystems are also highly spatially and temporally variable, so that regional systems are connected through ocean currents, ice drift and organism movement [9,23,24]. Trophic interactions are, therefore, scale-dependent, but there is little understanding how food-web structure varies across much of the polar oceans.

To predict the impacts of change, it is necessary to address these shortcomings and improve our understanding. To this end, we develop comparative analyses of large-scale variation in food webs in Arctic and Antarctic polar regions [25–27]. We focus on metazoan organisms in pelagic ecosystems, particularly in the epipelagic zone, to consider processes that link lower-trophic-level productivity to higher trophic level species across large areas of the polar oceans, concentrating particularly on the dominant role of a small number of individual species.

The following sections bring together the necessary elements for developing a consistent conceptual framework for understanding the interactions that determine ecosystem structure, functioning and resilience in polar ocean ecosystems. We briefly consider major determinants of food-web structure in these regions, how dominant food-web pathways vary across polar ecosystems and how these routes of energy flow operate as part of food-web networks. We explore variations in life-history strategies across these ecosystems and consider interactions of the biology of pelagic species and ecosystems that constrain species' success and food-web structures across scales. The conceptual framework we propose will help in the development of models and scenarios designed to project food web and ecosystem responses to change.

## 2. Polar ocean ecosystems: a comparative approach

### (a) Physical influences and productivity

The environmental influences at different scales on ecological processes in the polar oceans are generally understood [9,23,28]. Major differences between the two polar regions occur in the physical and primary production (PP) processes (summarized in the electronic supplementary material, table S1). The topography and circulation patterns account for much of the difference between the two polar regions, with strong north–south connectivity into and out of the Arctic, but circumpolar connectivity and greater isolation of the surface Southern Ocean [24]. The seasonal light climates also differ because the Southern Ocean does not extend towards the pole beyond approximately 78° S. Although the two regions differ in many aspects, there are broad similarities in the environmental structure of these polar systems, which we highlight to consider the ecological processes that determine the types of organisms that exist and their interactions (electronic supplementary material, table S1).

Both polar regions share general characteristics of low temperature, extreme seasonality (light climate) and the seasonal advance and retreat of sea ice. These characteristics set the basic environmental framework (electronic supplementary material, table S1) and produce intense periods of productivity that show general latitudinal gradients of seasonal development. Biological productivity in higher latitude regions is minimal for several months owing to little or no light [10]. Sea ice and associated snow cover reduce penetration of light into the upper ocean and hence the energy available to autotrophic organisms for production. However, microbial activity can be maintained in low light conditions, generating production (associated with ice-algae) within and on the under-surface of the ice [29]. Sea-ice retreat and melting produce shallow, stable mixed layers that result in favourable conditions for phytoplankton growth and marginal-ice-zone-associated blooms [30]. During spring and summer, intense phytoplankton blooms develop in open water regions, beginning earlier in areas farther from the poles and spreading towards higher latitudes as the ice retreats and irradiance levels and temperatures increase. As a result, bloom development in both polar regions is spatially and temporally variable [31,32] and is dominated by algal communities with specific adaptations to low light levels and temperatures [29].

Together, low temperatures and marked seasonality in primary productivity influence and potentially constrain intermediate trophic levels (micro, meso and macrozooplankton and nekton) that link lower and higher trophic-level species. Land-breeding seabirds and marine mammals are part of polar pelagic ecosystems, but require access to appropriate substrates for nesting or haul-out (land, sea ice or ice-shelves). The availability of these substrates and their proximity to appropriate food supply are important determinants of seabird and marine mammal distributions [33].

At both poles, physical conditions result in three major latitude-based habitat zones: (i) year-around sea-ice cover, (ii) seasonal sea ice, and (iii) open-ocean waters where sea ice rarely occurs [34,35]. Superimposed on these general characteristics are extensive regional heterogeneity and marked interannual and longer-term environmental variability [13,36].

### (b) Large-scale variation in polar ocean food webs

**Figure 1.**
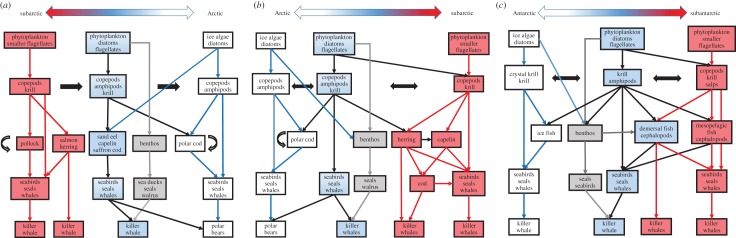
Major structural variations in polar marine food webs along a gradient (large double-headed arrow at top of panel) from the high-latitude sea-ice zone, through the seasonal-sea-ice zone to lower latitude open-ocean zones as a transect across the Arctic from the (*a*) subarctic Pacific to high Arctic, (*b*) from the high Arctic to subarctic Atlantic, and from the (*c*) high Antarctic to subantarctic, which are developed from [37] and based on a number of studies [9,13,16,38,39]. Environmental conditions are represented by advective fluxes (solid black single and double-headed arrows), sea-ice zone (white boxes and lines), seasonal sea-ice zone (blue boxes, black lines) and open-ocean zone (red boxes and lines). Benthic connections (grey boxes and lines), and cannibalism (black outlined arrow) are indicated. The different killer whale boxes represent sub-groups that specialize on fishes or marine mammals/flightless birds in different regions.

The diets of higher trophic level species in Antarctic and Arctic pelagic food webs highlight the general similarity of the systems, with the main flows involving few species and one or two trophic interactions from the lowest to the top-level species (figure 1). In the Arctic, variation in structure reflects the general latitudinal changes in habitat and production regimes from more open-ocean subarctic conditions through to high Arctic regions (figure 1*a,b*) [28,34,40]. Differences in trophic connection occur at mid-trophic levels and reflect the relative importance of a small number of copepod, euphausiid, amphipod and fish species. The dominant species at each trophic level relate to the main habitat zones noted above. The Pacific and Atlantic subarctic systems are similar in structure, but with regional differences (e.g. between the Bering–Chukchi and Greenland–Barents Sea) that reflect the degree of spatial connectivity, topography and influences from the adjacent Pacific or Atlantic Oceans (figure 1*a,b*) [13,25,37,38]. The same general zones characterize the main food webs of the Southern Ocean (figure 1*c*), although there is variation within zones associated with differences in productivity and species dominance.

Two major differences are apparent in the Arctic and Antarctic food webs [9,25,28,37,39]. First zooplankton–fish connections dominate in Arctic regions (figure 1*a,b*), whereas direct zooplankton–seabird and marine mammal pathways dominate in the Southern Ocean, with fish pathways of local importance (figure 1*c*). Second, benthic–pelagic interactions are more important in Arctic food webs (figure 1*b,c*) because of the extensive and relatively shallow shelf areas that surround the central ocean basin and adjacent southerly areas relative to the deeper continental shelves in the Southern Ocean (electronic supplementary material, table S1).

Exceptions to this general view arise because boundaries between habitat zones are not absolute; local ocean, ice and bathymetric conditions are often complex, and primary productivity varies, all of which can obscure broad-scale food-web structure. However, these synthetic food webs extend the traditional view of these ecosystems and show how the major energy flow pathways to higher trophic levels vary across both poles.

### (c) Alternative pathways in complex networks: interactions and variability

Detailed food-web analyses (ecopath models) are available for several polar regions and for this study, we considered three examples: the West Antarctic Peninsula (WAP) [41]; South Georgia shelf region [42] and the Barents Sea [43]. These examples span Antarctic, subantarctic and subarctic environments, respectively, and allow examination of the relative importance of different pathways in maintaining food-web structure in differing polar systems. For each system, a mass-balance model consumption-matrix was used to estimate the energy flows as a percentage of overall PP maintaining the food web (see the electronic supplementary material, figure S1). The ecological emphasis, species aggregation and data availability differed for each model implementation, but aggregation of the simulated data provides a simple general functional group/size-based view of the major food-web flows, thereby allowing comparison between systems (electronic supplementary material, figure S1). For the Southern Ocean food webs, flows through Antarctic krill (*Euphausia superba*) were considered separately (electronic supplementary material, figure S1*a,b*). The Barents Sea study focused on its southern region and particularly the various fish species that inhabit this area. For this analysis, a small/young fish category was considered separately as a general size-group between macrozooplankton and larger fishes (electronic supplementary material, figure S1*c*).

The short pathway through Antarctic krill dominates the consumption by the combined upper-trophic levels (fishes and larger species), supplying very similar levels (approx. 43%) of the demand for the WAP and South Georgia (approx. 44%, approx. 47% including off-shelf flows; electronic supplementary material, figure S1*a,b*). Krill contributes approximately 80% of the demand by seabird and marine mammal predators and approximately 41% by fish and cephalopods at the WAP, and approximately 65% of the consumption by seabirds and marine mammals and approximately 37% for fish and cephalopods at South Georgia. South Georgia receives substantial import of secondary production from off-shelf regions to support the shelf food web, and this occurs at similar proportions through the krill and non-krill pathways to that seen on the island's shelf (approx. 50 : 50).

Antarctic krill are the main prey species in both Antarctic systems, but other species of meso- and macrozooplankton, including copepods, amphipods and other euphausiid species are also important in energy flow to fishes and other larger species. Two fish species, Antarctic silverfish (*Pleuragramma antarcticum*) and lantern fish (*Electrona antarctica*), dominate the pelagic fish assemblage of the WAP system. At South Georgia, the mackerel icefish (*Champsocephalus gunnari*) is a krill consumer and myctophids are important in the diet of a range of predators. The dominance of the krill pathway at higher trophic levels is in contrast with the lower-trophic-level consumption of PP; only 2.8% of the WAP PP goes through krill and 5.0% at South Georgia.

In the Barents Sea, macrozooplankton and fish/cephalopods account for approximately 30% and approximately 66% of upper-trophic level consumption, respectively. The consumption of macrozooplankton consists mainly of euphausiids (particularly *Thysanoessa inermis, Thysanoessa raschii* and *Meganyctiphanes norvegica*, an expatriate from the Norwegian Sea) and amphipods (particularly the ice-associated *Themisto libellula*), but also includes fish larvae [8,25,37,43]. The fish species consume mesozooplankton, particularly *Calanus glacialis*, *Calanus hyperboreous* and *Calanus finmarchicus* (also an expatriate from the Norwegian Sea) (of these, herbivorous zooplankton constitute 60% and carnivorous zooplankton 40%). For the higher predators, consumption of fish, especially herring (*Clupea harengus*), but also capelin (*Mallotus villosus*), cod (*Gadus morhua*) and haddock (*Melanogrammus aeglefinus*) are all important [16]. The Barents Sea analysis (electronic supplementary material, figure S1*c*) indicates that benthic coupling is more important (total flows from benthos into food web approx. 8% of PP) than at the WAP and South Georgia (total flows <4% of PP into food web), but we note that benthic interactions were better resolved in the original Barents Sea study.

As noted above (§2b), there is marked variation in the species composition across the polar regions, so each of the food-web analyses considered above can only provide a regional snapshot. More extensive comparative analyses across more ecosystems are required, and the available information from other studies indicates similar complexity exists in other regional ecosystems. Analyses of Arctic regional food webs in the Chukchi Sea, Eastern Bering Sea, Gulf of Alaska and the Barents Sea indicate that energy flows to higher trophic levels are dominated by particular pathways, and also highlight the importance of benthic–pelagic and wider food-web interactions [27,43]. In the highest latitude shelf regions of the Southern Ocean, such as the Ross Sea, two species are particularly important in the diets of the higher predators, crystal (or ice) krill (*Euphausia crystallorophias*) and Antarctic silverfish, but Antarctic krill remain important in slope areas [22,39,44]. Although there are important gaps in information on food-web processes for key areas, such as the central Arctic Basin or the Weddell Sea, the traditional view of a dominant pathway maintaining upper-trophic levels in polar pelagic ecosystems is supported by the available syntheses. These dominant pathways are part of a larger network that includes important alternative pathways, which also involve a restricted set of trophic interactions [9,12,13,16].

The expression of alternate trophic pathways is mediated by variability in environmental conditions (e.g. oceanic and sea-ice conditions) affecting regional productivity and food-web interactions. The Antarctic Peninsula and South Georgia undergo large interannual changes in the relative abundance of Antarctic krill and associated zooplankton community composition [26,36]. Fluctuations in krill abundance generates redirection of energy flows throughout the food web [9]. Predators consume different prey species, using particular alternative pathways of energy flow, thereby providing a buffer in the overall food-web structure that allows adult predators to survive. Similar effects of interannual variability on the relative dominance of different trophic interactions have been observed in Arctic food webs (e.g. [13,21,45]).

Seasonal changes in feeding also involve alternative pathways of energy flow, the exact nature of which depends on the species and locality [9,17,37]. Lower-trophic-level activity continues during winter through recycling and food-web interactions in even the highest latitude polar regions [46]. These seasonal changes in food-web operation often result in a shift to a higher trophic level of feeding during winter. For the upper-trophic levels, this reflects a general shift from feeding primarily on herbivorous crustaceans during summer to carnivorous zooplankton and fishes in winter. Many of the major predator species disperse or move away during winter, reducing local demand and mortality and connecting food webs in different ecosystems [22,23,26,36,42].

### (d) Sub-system and spatial connections

Understanding process interactions across scales in polar ecosystems is fundamental to predicting impacts of change [23,38]. Important connections between sub-systems (e.g. ice-ocean, benthic–pelagic) and spatially separate sub-systems contribute to maintenance of the whole ecosystem. Food-web interactions between surface waters and deeper regions are important in both poles. These interactions are a major feature of Arctic shelf systems where ice-associated production, local pelagic blooms and advected production maintain development of extensive and rich benthic communities and support larger air-breathing predators (e.g. walrus and bearded seals) [16,27].

Although not a unique characteristic, advection and organism movement are considered important influences on the structure and functioning of Southern Ocean and Arctic ecosystems [9,16,24,38,47]. Horizontal advective fluxes connect regional systems by moving production from areas of generation to areas of consumption, thereby disconnecting production from consumption/export processes [9]. Organisms transported into unfavourable habitats may be unable to successfully reproduce or grow, but can still be major components of local food webs, for example, *M. norvegica* and *C. finmarchicus* in the northern Barents Sea, and Antarctic krill in northern areas of the Scotia Sea [36,38]. As a result, local and regional abundances of some key pelagic species in polar food webs are determined by factors that influence the magnitude of advective fluxes, making them vulnerable to environmental change.

## 3. The importance of individual species

Polar oceans support complex microbial systems but pelagic ecosystems are generally lower in metazoan diversity than other oceanic systems [48,49]. This is part of a more general, and much debated, macro-ecological relationship of decreasing diversity of biological communities with increasing latitude [48], and provides the biodiversity context within which the ecosystems operate. Only a few species dominate the pelagic energy flows in polar food webs. Here we consider life histories of these dominant pelagic species to examine what constitutes a successful strategy, focusing particularly on zooplankton that are crucial in mid-trophic levels.

### (a) Life histories of polar species

In comparison with lower latitudes, polar pelagic species tend to have slower growth rates and extended or multi-year life cycles (see e.g. [50,51]). Many key species also have complex life-cycle strategies, which include reduction or cessation of metabolic processes, build-up of energy stores (fatty acids and lipids), switching diet preferences (e.g. herbivorous to carnivorous, specialist to generalist), and access to alternative food sources (e.g. benthic or sea-ice associated feeding) or migration into other ecosystems during certain life-cycle stages where food is available or costs can be reduced (e.g. [33,37,50]). The dominance of a small number of species in polar pelagic food webs means that understanding their life-history strategies and associated traits is fundamental in analyses of trophic interactions.

The two main zooplankton groups in food webs in both polar regions are the mesozooplankton calanoid copepods (adults 3–7 mm) and the macrozooplankton euphausiids (adults 25–60 mm) [50–52]. In both groups, species are large compared with other members of the same genera at lower latitudes. The importance of a few species of copepods and euphausiids in polar marine food webs, which can also have similar feeding modes and hence trophic levels, makes differences in their traits (including their relative size) and abundance important influences on food-web structure and ecosystem functioning [9,16,26].

**Figure 2.**
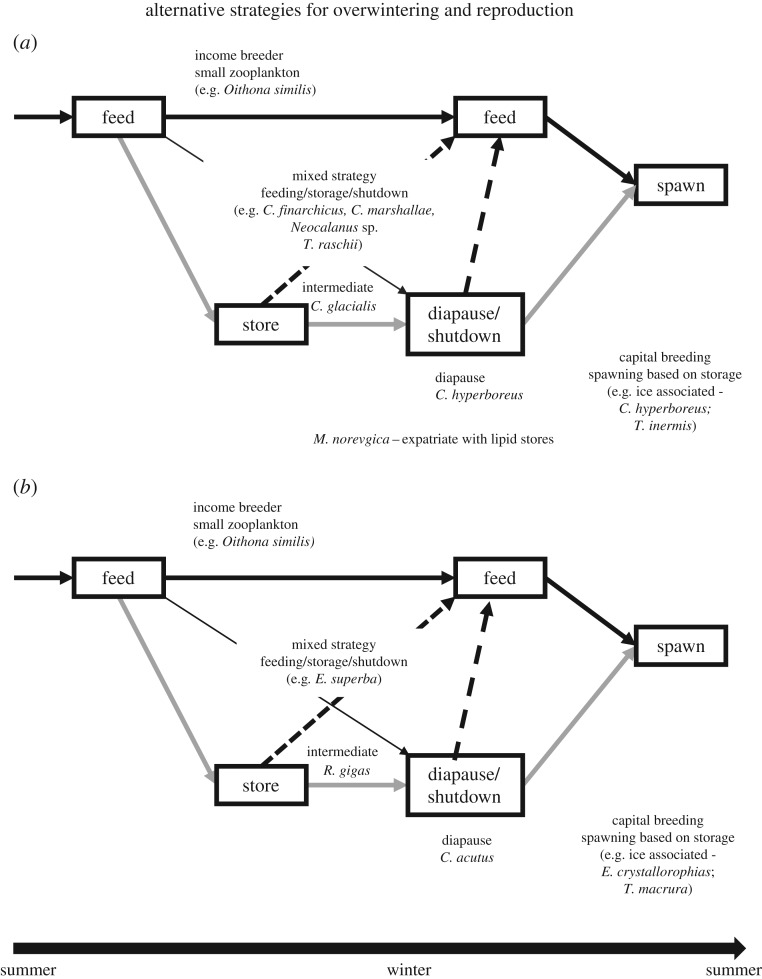
Schematic view of alternative life-history strategies for overwintering and reproduction of (*a*) Arctic and (*b*) Antarctic zooplankton [50,51,53–56] with general properties of some of the key species of meso- and macrozooplankton (see text) in polar ocean ecosystems highlighted.

Zooplankton species at both poles adopt a range of sub-strategies as part of their overall life cycle. The copepod and euphausiid strategies suggest that there are analogue species in the two systems [50–55] (figure 2). The main Arctic under-ice zooplankton species, *C. hyperboreus*, has a capital breeding/spawning strategy based on lipid stores (figure 2*a*) similar to that of *T. inermis* and its larger Antarctic analogue the crystal krill, *E. crystallorophias* (figure 2*b*). This allows the larvae of these species to use early season ice-associated production to grow and develop. By contrast, *C. glacialis* in the Arctic (figure 2*a*) and *Calanoides acutus* and *Rhincalanus gigas* in the Antarctic (figure 2*b*) generally adopt a shutdown and diapause strategy. *Calanus glacialis* can reproduce using lipid reserves in ice-covered regions by feeding in open-ocean regions. The subarctic *C. marshallae* in the Bering Sea and *C. finmarchicus* in the Norwegian–Barents seas (figure 2*a*) have a mixed strategy of feeding, storage and shutdown, with a dependence on the spring bloom for reproduction, which is similar to that of the euphausiid, *T. raschii*, found in the Barents, Bering and Chukchi/Beaufort seas and that of Antarctic krill (figure 2*b*). In both polar systems, there are smaller species that have mixed strategies and can feed throughout the year, such as the ubiquitous copepod *Oithona similis*, which is an income-based breeder (reproduction fuelled by consumption) at both poles.

The life-history strategies of polar zooplankton (figure 2) indicate relatively few successful strategies and an evolutionary convergence across organisms that are an order of magnitude different in size. This may not be unique to the polar oceans, but suggests a conceptual view of alternative life-history strategies of polar zooplankton that has three main elements: (i) continuous feeding and reproduction (income breeders), (ii) lipid storage and diapause, and (iii) extended life cycles, which can involve delayed development and maturation. This generalized view also suggests that copepods (from small to large) and the euphausiids, including Antarctic krill, can be represented in such a continuum. This continuum provides a potential framework for developing alternative models for polar zooplankton that are based on optimal life histories from which species types should emerge under different conditions. This type of model structure has been considered for Arctic copepods [57], and it may be generally applicable to polar zooplankton.

A small number of Arctic fish species, such as polar cod (*Boreogadus saida*) in high Arctic regions, juvenile walleye pollock (*Gadus chalcogrammus*) in the Bering Sea, and herring and capelin in the Atlantic-influenced regions of the Barents Sea, dominate pathways that link copepods and higher predators [25,27,37]. Polar cod have a life cycle closely associated with sea ice, while the subarctic species occur mainly in shelf areas influenced by current flows from lower latitudes [21]. Large epipelagic fish species are generally absent in the Southern Ocean except in the most northern regions [17,24,39]. In the Antarctic continental shelf areas, where sea ice is important, a single fish species, Antarctic silverfish dominates, which has a life cycle that is closely associated with sea ice [22,39]. Antarctic fish species are mainly from a single family, the Notothenioids, and have a generally demersal or semi-demersal habit. Mesopelagic fishes are generally small (10–20 cm), and are important consumers and prey in Southern Ocean pelagic and mesopelagic food webs [41,42], but are less important in the shallow shelf areas of much of the Arctic and absent from the central Arctic Ocean. Squid are also present and appear to generally operate as mesopelagic species, although they may be most abundant in the Antarctic polar frontal region. Their abundance, biomass, life histories, diet and prey demand are poorly known [42].

Seabirds and marine mammals have two basic strategies: staying or moving during winter [24,33,58]. Species that remain in polar regions (e.g. ice-dependant seals and some seabirds) may disperse across increased areas of sea-ice habitat, potentially reducing local competition. Those that leave during winter may undertake extensive migrations to more favourable areas, including the opposite pole. Within the stay/move strategies there are examples of organisms that continue to actively feed and others that stop or greatly reduce feeding, becoming more dependent on energy stores laid down during summer. The composition of the diet, fatty acid and lipid reserves in these organisms is important in migration, overwintering and spring reproduction [59].

Storage of energy and/or the reduction of metabolic costs during winter are a defining characteristic of larger plankton, nekton and higher trophic-level species in polar ecosystems [8,52,55,59]. The ubiquity of lipids in the life histories of polar species suggests that the evolutionary development of lipid biochemical processes was an important influence on current biodiversity of polar ocean ecosystems. Zooplankton have developed life cycles that are strongly dependent on the acquisition of fatty acids and lipids to fuel growth and development and to provide energy stores for overwintering. Some polar species have also developed the capacity to biosynthesize lipids with a higher energy capacity [51,55]. These high-energy molecules are crucial in the diets of many of the high-latitude fish, seabird and marine mammal species, providing them with the energy reserves required to withstand extended periods of low food availability or undertake migrations, and hence are also important in food-web processes [52,56,60,61].

## 4. Discussion and conclusion

### (a) Pelagic ecosystem structure and functioning

Our comparative analyses of the structure of pelagic ecosystems in polar oceans show that the traditional view of a relatively simple ecosystem with short pathways is appropriate for metazoan organisms. However, systematic changes in food webs that are strongly constrained by regional habitat characteristics allow different species to dominate the main pathways of energy flow. This is most clear in the high Antarctic, where crystal krill and silverfish dominate the energy flows in ice-covered regions; by contrast, Antarctic krill are the main prey in the somewhat lower latitude more productive regions [9,39]. In high-latitude ice-influenced regions of the Arctic, ice-associated copepods *C. glacialis*, *C. hyperboreus*, the amphipod *Themisto libellula* and polar cod dominate energy flows [25,27]. Farther south in the Arctic, other copepod and fish species are important, but these differ in the Atlantic and Pacific regions. This integrated perspective, which relates food-web structure strongly to habitat, suggests that large-scale changes in the major structural and functional characteristics of pelagic ecosystems in both the Arctic and Antarctic are potentially predictable.

The operation of the dominant and alternative pathways of energy flow as part of more complex food-web networks highlights important aspects of how species composition and ecosystem processes interact in polar pelagic ecosystems. Ecosystems with restricted energy flow to higher trophic levels through one or two mid-trophic-level species occur throughout the polar oceans. The individual metazoan species involved have a disproportionately large effect on ecosystem functioning, emphasizing that these systems have low functional redundancy [3,19]. This structure places dynamic controls on bottom-up and top-down food-web processes that select for important alternative pathways of energy flow that involve particular sets of trophic interactions. Fluctuations in the abundance of key species allow expression of these alternative pathways [9,13,16,17,21,22], which are often weaker and less efficient in transferring energy to the highest trophic levels. These different routes provide important alternative energy sources during winter and periods when dominant pathways fail [13,38,49]. Food-web interactions (including magnitudes) and structure that emerges in response to seasonal and interannual variation are, therefore, important in maintaining ecosystem productivity and overall resilience properties [3,12]. Although these alternative, more complex pathways help the system absorb short-term fluctuations, they probably cannot substitute in the longer term (years to decades) for the short, high-energy, flow routes that maintain the highest trophic-level species [9,13]. Loss of the key mid-trophic species is likely to lead to reductions in higher trophic-level abundance because there are so few species that can occupy the same role [9,13,26].

The distribution and abundance of key polar species and their traits are fundamental determinants of food-web structure. For example, the large size of Antarctic krill, its omnivorous feeding strategy and its propensity to form schools or swarms allows efficient energy transfer from low to high trophic levels, and is key to maintaining the high biomass of large predators in the Southern Ocean [9]. Antarctic krill has a complex life cycle that is strongly linked to sea ice, which supports larval and adult overwintering, and water mass structure that is critical to spawning and development of early life stages [9,47]. Thus, detailed analyses of its life cycle processes (and adaptive capacity) are critical to predicting how change will impact this key species. The key role of polar cod in the Arctic food webs and its life history provides a northern latitude example [37]. Life histories and food-web processes are not separate and, in polar pelagic ecosystems where individual species can dominate and there is low functional redundancy, the focus needs to be broader than on a single aspect of either.

### (b) A new conceptual framework for analyses of polar pelagic ecosystems

**Figure 3.**
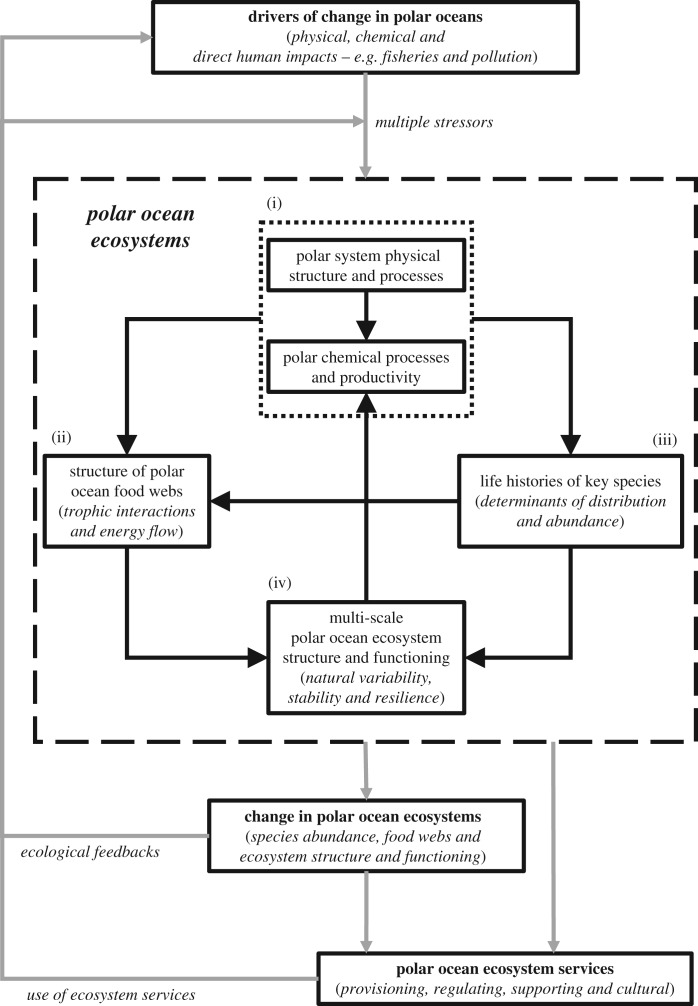
A conceptual framework for analyses of the determinants of polar pelagic ecosystem structure and functioning and the impacts of change. Understanding the impacts of multiple drivers of change requires analyses of food webs and key species life histories across multiple scales (large dashed-line box; ecosystem components (*i* to *iv*); connected by black arrows). These ecosystems maintain services that are also affected by ecosystem changes. Ecosystem changes and use of services feedback to further impact the ecosystems (grey arrows indicate change and ecosystem service connections).

With this view, we propose a conceptual framework (figure 3) for analyses of the determinants of ecosystem structure and functioning that integrates the aspects we have highlighted: (i) physical and chemical environmental influences, (ii) detailed structure of food webs, (iii) quantified understanding of key (functional) species' life histories and adaptive capacities, and (iv) analyses of ecosystems across scales. The underlying physical and chemical system (i; §2a) sets the basic habitat and productivity regime within which species operate and interact in a food-web network (ii; §2b). The main energy flows are maintained along a small number of dominant and alternative pathways (ii; §2b) that involve a small set of key species whose relative success is determined by their life histories and associated traits, and which influences food-web structure (iii; §3). The final component of this framework (iv) requires understanding and integrated analyses of ecosystem functioning, key species life cycles and food-web processes across a range of spatial and temporal scales [9,21,23].

This framework is a preliminary step towards the development of a consistent approach to modelling the interactions that determine ecosystem structure and functioning that can be used to assess and project the impacts of future change in polar ocean ecosystems and on ecosystem services (figure 3). This approach requires extensive field and laboratory studies to address major gaps in understanding of species interactions, and life cycles and adaptive capacities and of wider ecosystem processes. Moreover, the ability to develop quantitative models in polar systems is limited by basic understanding of the links between sea ice and pelagic systems, pelagic and benthic systems and seasonal changes in trophic interactions. To improve understanding of the structure and functioning of polar pelagic ecosystems, a systematic and quantified approach is required to generate analyses of the seasonal operation of food webs and key species' life cycles within and across polar systems, along with a comprehensive assessment of the functional diversity of polar pelagic species. Our analysis focused on the flow of energy to higher trophic levels, only one of the many dependencies regulating ecosystem functioning. Detailed information is required on the relative importance of different trophic interactions (including under-represented species) in overall ecosystem functioning. For example, the effects of shifting phytoplankton and zooplankton assemblages associated with changing ice cover on food-web processes and related biogeochemical cycles are not known, but are projected to occur as both polar regions change. The desire to understand, quantify and predict these changes highlights the need for more comprehensive analyses of the biological and functional diversity of polar pelagic ecosystems.

### (c) Implications for understanding impacts of change

An obvious implication of warming is that environmentally driven poleward shifts in the major habitat boundaries will result in changes in pathways of energy flow that dominate regional food webs. Changes in the spatial pattern of food-web structure and functioning are unlikely to be simple, as alternative pathways of energy flow involve species that have different sensitivities to environmental perturbations. The strong dependence of polar systems on a small number of species with highly specialized life cycles and different adaptive capacities suggests that projections of impacts of change should resolve the dynamics of species functional roles as well as levels or timing of productivity and the responses of aggregated functional groups.

Projected warming and sea-ice reductions for both poles over the next century could potentially produce a shift in sub-polar regions from a planktonic community dominated by large, lipid-rich copepod and euphausiid species to one that is more diverse with smaller zooplankton. Under such a scenario, successful species would have different phenologies and lipid characteristics, and carnivorous zooplankton may become more important as prey for larger organisms. Such an ecosystem, involving different and more complex pathways of energy flow, would not support the present abundance of large predators, unless overall system productivity increases. Change in bloom timing and lipid biochemistry may disrupt life-cycle and overwintering processes of mid-trophic and upper-trophic level species. Some of the key mid-trophic level species may not be able to complete their life cycles at latitudes characterized by different ocean circulation patterns, shelf structure and production cycles. This may result in tipping points being reached that produce rapid changes in abundance and distribution of key species, and hence in regional food webs and wider ecosystem structure and functioning.

Developing an understanding of how species composition, and biodiversity more generally, influence ecosystem functioning will, therefore, require a mechanistic understanding that goes far beyond that currently available in most polar regions [12,23,28,46]. Observational systems aimed at detecting change in these regions need to encompass aspects of both biodiversity and ecosystem structure and functioning. Understanding and projecting impacts of change in these important ecosystems requires integrated approaches that combine analyses and model development of life cycles and functional roles of key species (including adaptive capacities), food-web processes and dynamics, and environmental controls over a wide range of space and time scales. Only through this approach can the range of potential impacts and responses be assessed, and valuation frameworks developed for ecosystem services. This will be important for the development of effective policies and management strategies for these vulnerable polar ecosystems.

## Supplementary Material

Supplementary Information
