# Supplementary material for: Understanding the structure and functioning of polar pelagic ecosystems to predict the impacts of change
Source: Proc Biol Sci. 2016 Dec 14;283(1844):20161646. doi: 10.1098/rspb.2016.1646 (PMC5204148; doi:10.1098/rspb.2016.1646)
Supplement: Supplementary Information [file rspb20161646supp1.pdf]

Supplementary information:

E.J. Murphy, R. D. Cavanagh, K. F. Drinkwater, S. M. Grant, J. J. Heymans, E. E. Hofmann, G. L. Hunt, Jr. and N.M. Johnston

Understanding the structure and functioning of polar pelagic ecosystems to predict the impacts of change

Proceedings of the Royal Society–B Biological Sciences
doi: 10.1098/rspb.2016.1646

Supplementary information.

Supplementary Table S1 and Figure S1 with associated explanatory material and references.

Supplementary Table S1 Environmental characteristics of the Arctic Ocean and Southern Ocean that are important influences on metazoan pelagic biodiversity and ecosystem structure and functioning [1, 2].

Feature	**Arctic Ocean**	**Southern Ocean**
Topography [1]	Almost completely surrounded by land, occupying the high-latitude polar region and is connected north-south to both the Pacific and Atlantic Oceans.<br><br>The continental shelf areas of North America and Europe are extensive and generally shallow (<100 m), while the central Arctic Basin is 2000 to 4000m deep.	Continuous water mass surrounding the Antarctic continent links all of the world's major oceans.<br><br>Deep continental shelf areas (> 500m). Submarine mountainous arcs extend to the surface and island archipelagos occur with locally extensive shelf regions. Generally greater than 2000 m deep.
Major currents and structure [1]	The northward flowing Norwegian Atlantic Current between Greenland and Norway brings Atlantic waters into the Barents Sea region and shelf areas to the east. Farther west, there is the southward flowing East Greenland Current, which brings polar waters south to areas around southern Greenland and further west towards the Canadian coast.<br><br>On the Pacific side, exchanges with the Arctic are important but more restricted by the shallow (~50 m) Bering Strait, which separates the Bering Sea to the south from the Chukchi Sea to the north.	The Antarctic Circumpolar Current (ACC) flows clockwise around the continent. In areas south of the ACC, there are a series of clockwise flowing large gyres (Weddell Sea and Ross Sea), while close to the continent westward flows occur in association the Antarctic Coastal Current.<br><br>Sub-Antarctic Front and Polar Frontal Zone acts as a boundary to north-south flows in the upper ocean, generally isolating these waters from areas farther north.<br><br>Circumpolar deep water upwells at the Southern Boundary of ACC and in restricted areas of continental shelf, bringing warm high nutrient waters to the surface.
	Almost the whole of the region to the north of 80°N is oceanic [1].<br><br>Much of the Arctic basin is ice covered and in complete darkness for several months;<br><br>Sea ice extends equatorward of 60° latitude in limited regions.	There are no oceanic regions south of ~78°S.<br><br>The highest latitude habitat is not present in the Southern Ocean.<br><br>Sea ice extends equatorward of 60° over large areas.
Production [3] [4]	Most of the production in ice and open water is fuelled by algal growth, with communities dominated by species that have specific adaptations to the low light levels, temperatures and salinities. Blooms occur in marginal ice zone and open water regions.**<br><br>Intense blooms dominated by diatom species.<br><br>Where conditions for growth are less favourable, production is generally low and smaller autotrophs (diatoms and dinoflagellates) dominate the plankton communities.**	**<br><br>Intense blooms dominated by diatom species. The prymnesiophyte, *Phaeocystis,* can also be important.<br><br>**<br><br>Light and iron are the main limiting factors [6].

	Production generally constrained by light and macronutrient availability.<br><br>Where there is little snow cover during spring, light penetration through the ice can generate large early season blooms below the ice [5].	In the Southern Ocean, iron is crucial for bloom development, generating extensive and sustained blooms in areas high in macronutrients in continental shelf waters south of the Southern Boundary of the ACC [7] and around various Southern Ocean archipelagos [8].
Substrate for breeding seabirds and marine mammals	Require access to appropriate substrates for nesting, or haul-out while ice-obligate species haul-out on sea-ice or breed on ice-shelves. The availability of these conditions and their vicinity to appropriate food supply limits the potential distribution of these species [9].**	**
Variability	Marked interannual variability in ocean and ice conditions associated with hemisphere scale atmospheric variability (e.g. North Atlantic Osscillation (NAO) and El Niño Southern Oscillation (ENSO). Regional footprint varies throughout the Arctic [10].	Marked interannual variability in ocean and ice conditions associated with hemisphere scale atmospheric variability (e.g. Southern Annular Mode (SAM) and ENSO). Regional footprint varies throughout the Southern Ocean [10].
Change	Overall reduction in winter sea ice extent.<br><br>Increased influence of North Atlantic waters in Barents Sea region [10].	Small overall increase in circumpolar winter sea-ice extent.<br><br>Large regional differences. Increases in Ross Sea region and decreases around West Antarctic Peninsula [10].

**Statements apply to Arctic and Antarctic

Supplementary Figure S1 Consumption flows derived from Ecopath mass balance models constructed for: (a) west Antarctic Peninsula [11], (b) South Georgia in the northern Scotia Sea [12] and (c) the Barents Sea [13]. The food webs were derived using the balanced consumption matrix from each model and flows aggregated into the groups shown. Flows are expressed as percentages of the total primary production supporting the pelagic food-web. Benthic flows are shown separately and expressed as percentage of the total pelagic production. The coloured lines indicate the source group for flows.

The three studies analysed the food webs at very different levels of taxonomic or functional resolution. As far as possible we mapped the available information onto the groups shown, but this was not always simple and required some interpretation based on the information presented in the individual studies. The exact flow rates do change based on the aggregation decisions, but the general pattern, relative magnitudes and routes of energy flow are not sensitive. For some flows disaggregation into the required structure was not possible so aggregated flows are illustrated. For example, the Barents Sea model does not distinguish meso- and macrozooplankton so the associated flows are combined in the food web figure (Figure S1c). Numerical values of combined fluxes are shown within an outlined box – the outline colour indicates the combined source. Focusing on the trophic flow between boxes and connections to the highest trophic levels, we do not show most return flows and internal box consumptions (e.g. fish eating fish). The original studies show the more detailed interactions in each system.

For comparative purposes the direct phytoplankton flux into the benthos in the Barents Sea model was excluded from the calculation of the total pelagic primary production required to support the food web. The direct flux was accounted for in the calculation of overall benthic fluxes. Detrital and benthic flows were aggregated and are shown as total inputs and outputs. Flows into the detrital and benthic pools were distinguished in the west Antarctic Peninsula and South Georgia models. For the Barents Sea the benthic system components and associated flows were analysed at much finer resolution, but detrital flows were not fully distinguished. For comparative purposes aggregated flows in a combined detrital and benthic pool are shown, while pelagic food web associated flows involving benthic organisms are shown as a separate set of aggregated flows (purple arrows and outline). The South Georgia model included two offshelf source boxes representing import/influx of i. krill and ii. other zooplankton or fish. For the calculation of relative consumption from different sources shown in the text, we totalled all inputs and calculated percentages of the consumption from different sources (including benthic and internal box consumptions (e.g. fish eating fish). These flows are not shown on the figures.

The model analyses undertaken here are preliminary and illustrate major pathways of flow in relation to food web structure. Further analyses with improved resolution of the trophic interactions and flows are required to develop consistent comparative studies, ideally based on the same level of taxonomic or functional group resolution [14]. The extent to which such data exists for different regional systems varies. Development of consistent high resolution analyses and datasets of trophic interaction for different regional systems are needed to improve comparative analyses of food web structure. This will require dedicated food web studies with standardised approaches to sample collection and analyses.

References

1. Hunt G.L., Drinkwater K.D., Arrigo K., Berge J., Daly K., Danielson S., Daase M., Hop H., Isla E., Karnovsky N., et al. in review Advection in polar and sub-polar environments: impacts on high latitude marine ecosystems. *Prog Oceanogr*.
2. Wassmann P., Kosobokova K.N., Slagstad D., Drinkvvater K.F., Hoperoft R.R., Moore S.E., Ellingsen I., Nelson R.J., Carmack E., Popova E., et al. 2015 The contiguous domains of Arctic Ocean advection: Trails of life and death. *Prog Oceanogr* **139**, 42-65. (doi:10.1016/j.pocean.2015.06.011).
3. Smetacek V., Assmy P., Henjes J. 2004 The role of grazing in structuring Southern Ocean pelagic ecosystems and biogeochemical cycles. *Antarct Sci* **16**(4), 541-558. (doi:10.1017/s0954102004002317).
4. Arrigo K.R. 2014 Sea Ice Ecosystems. *Ann Rev Mar Sci,* **6**, 439-467. (doi:10.1146/annurev-marine-010213-135103).
5. Arrigo K.R., Perovich D.K., Pickart R.S., Brown Z.W., van Dijken G.L., Lowry K.E., Mills M.M., Palmer M.A., Balch W.M., Bahr F., et al. 2012 Massive Phytoplankton Blooms Under Arctic Sea Ice. *Science* **336**(6087), 1408-1408. (doi:10.1126/science.1215065).
6. Smetacek V., Nicol S. 2005 Polar ocean ecosystems in a changing world. *Nature* **437**(7057), 362-368.
7. McGillicuddy D.J., Jr., Sedwick P.N., Dinniman M.S., Arrigo K.R., Bibby T.S., Greenan B.J.W., Hofmann E.E., Klinck J.M., Smith W.O., Jr. , Mack S.L., et al. 2015 Iron supply and demand in an Antarctic shelf ecosystem. *Geophys Res Let* **42**(19), 8088-8097. (doi:doi:10.1002/2015gl065727).
8. Murphy E.J., Watkins J.L., Trathan P.N., Reid K., Meredith M.P., Thorpe S.E., Johnston N.M., Clarke A., Tarling G.A., Collins M.A., et al. 2007 Spatial and temporal operation of the Scotia Sea ecosystem: a review of large-scale links in a krill centred food web. *Phil Trans Roy Soc B* **362**(1477), 113-148. (doi:10.1098/rstb.2006.1957).
9. Tynan C., Ainley D.G., Stirling I. 2010 Sea ice: a critical habitat for polar marine mammals and birds. In: *Sea ice* (eds. Thomas D.N., Dieckmann G.S.), pp. 395-423. Oxford, Wiley-Blackwell.
10. Turner J., Marshall G. 2011 *Climate Change in the Polar Regions*. Cambridge, Cambridge University Press; 448pp p.
11. Ballerini T., Hofmann E.E., Ainley D.G., Daly K., Marrari M., Ribic C.A., Smith W.O., Jr., Steele J.H. 2014 Productivity and linkages of the food web of the southern region of the western Antarctic Peninsula continental shelf. *Prog Oceanogr* **122**, 10-29. (doi:10.1016/j.pocean.2013.11.007).
12. Hill S.L., Keeble K., Atkinson A., Murphy E.J. 2012 A foodweb model to explore uncertainties in the South Georgia shelf pelagic ecosystem. *Deep-Sea Research Part II-Topical Studies in Oceanography* **59**, 237-252. (doi:10.1016/j.dsr2.2011.09.001).
13. Blanchard J.L., Pinnegar J.K., Mackinson S. 2002 Exploring marine mammal–fishery interactions using ‘Ecopath with Ecosim’: modelling the Barents Sea ecosystem. In: *Science Series Technical Report* (p. 52 pp. CEFAS, Lowestoft, UK
14. Whitehouse G.A., Aydin K., Essington T.E., Hunt G.L., Jr. 2014 A trophic mass balance model of the eastern Chukchi Sea with comparisons to other high-latitude systems. *Polar Biol* **37**(7), 911-939. (doi:10.1007/s00300-014-1490-1).

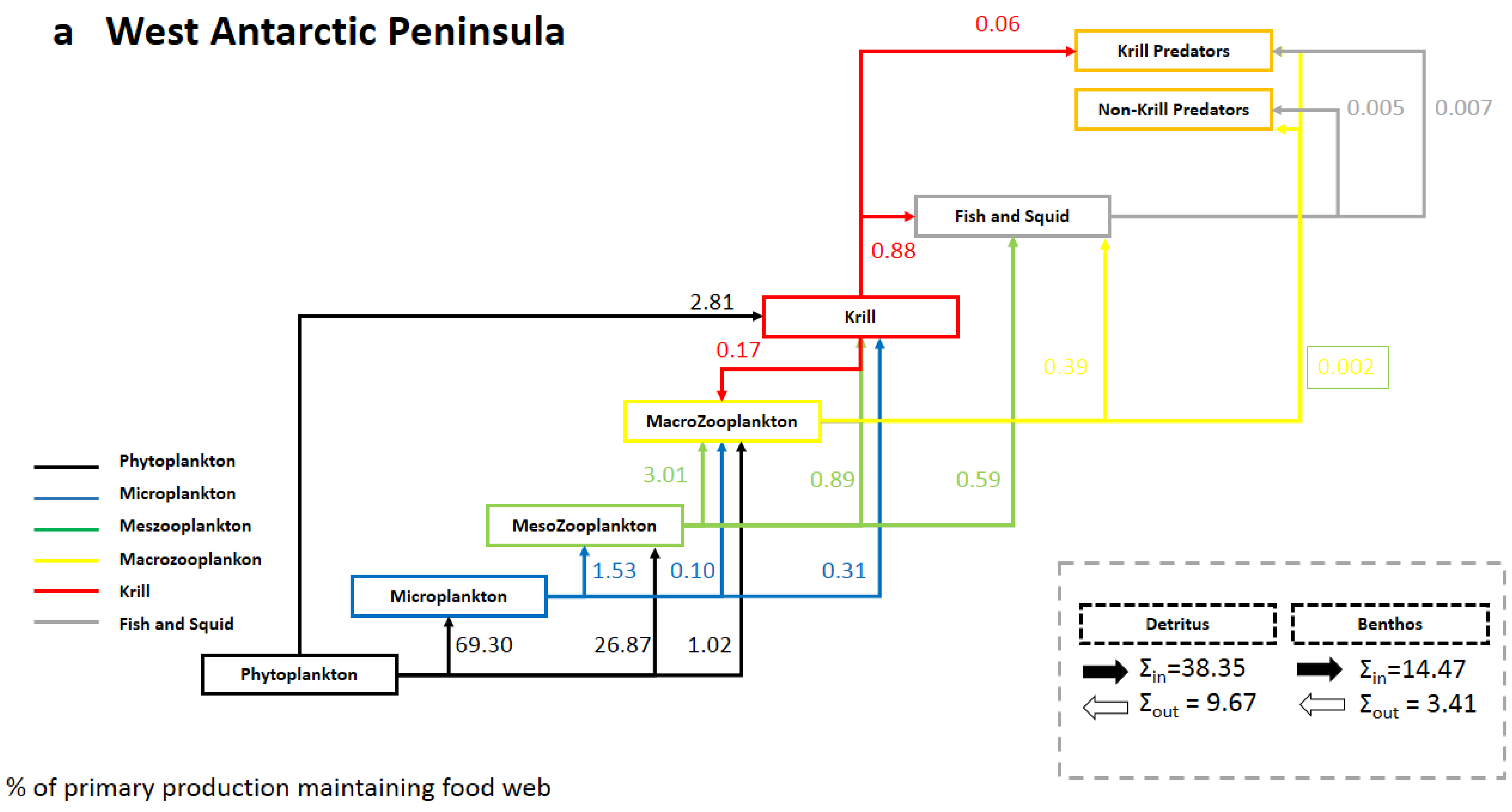

Supplementary Figure S1

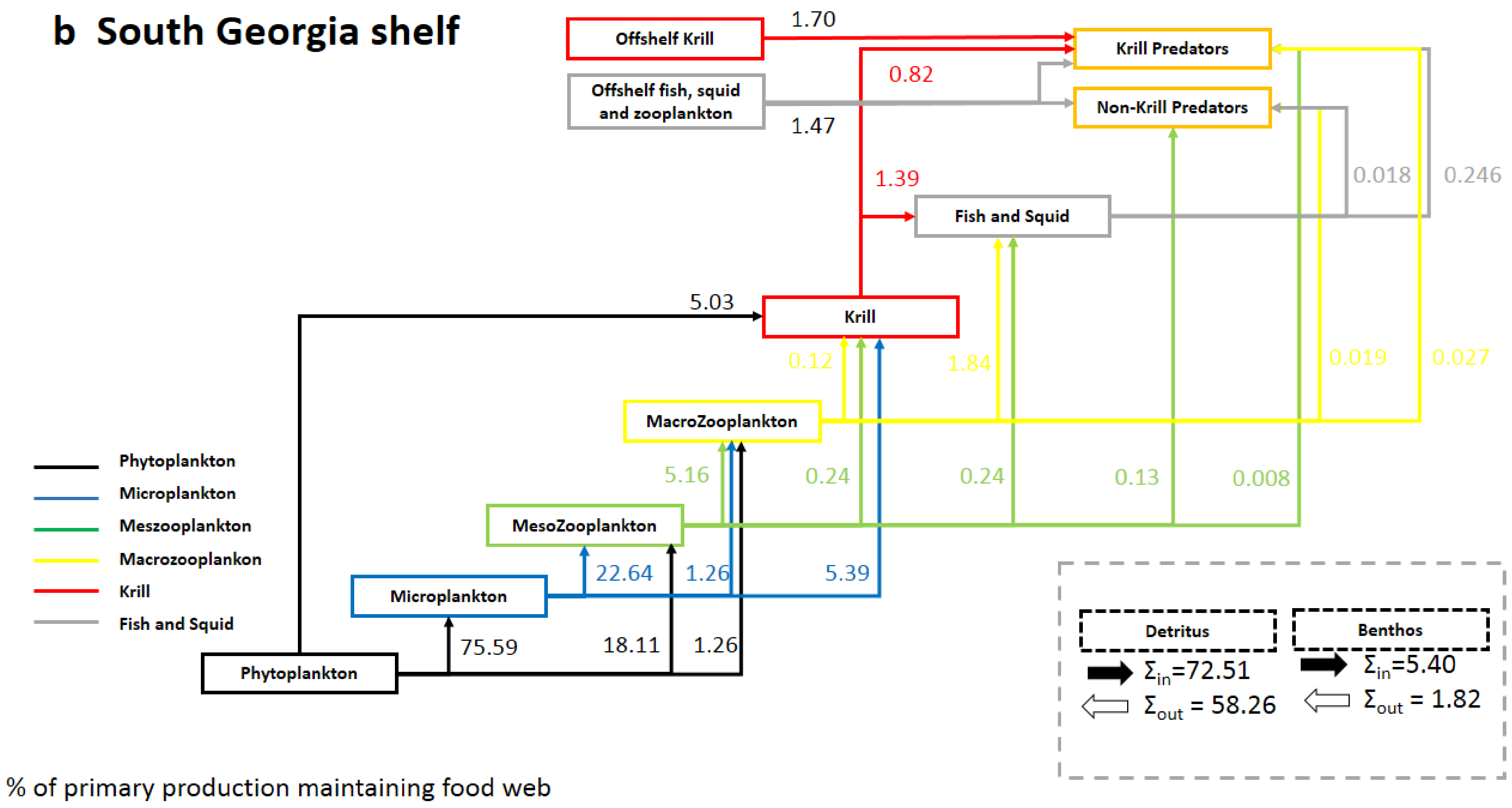

Supplementary Figure S1

Supplementary Figure S1

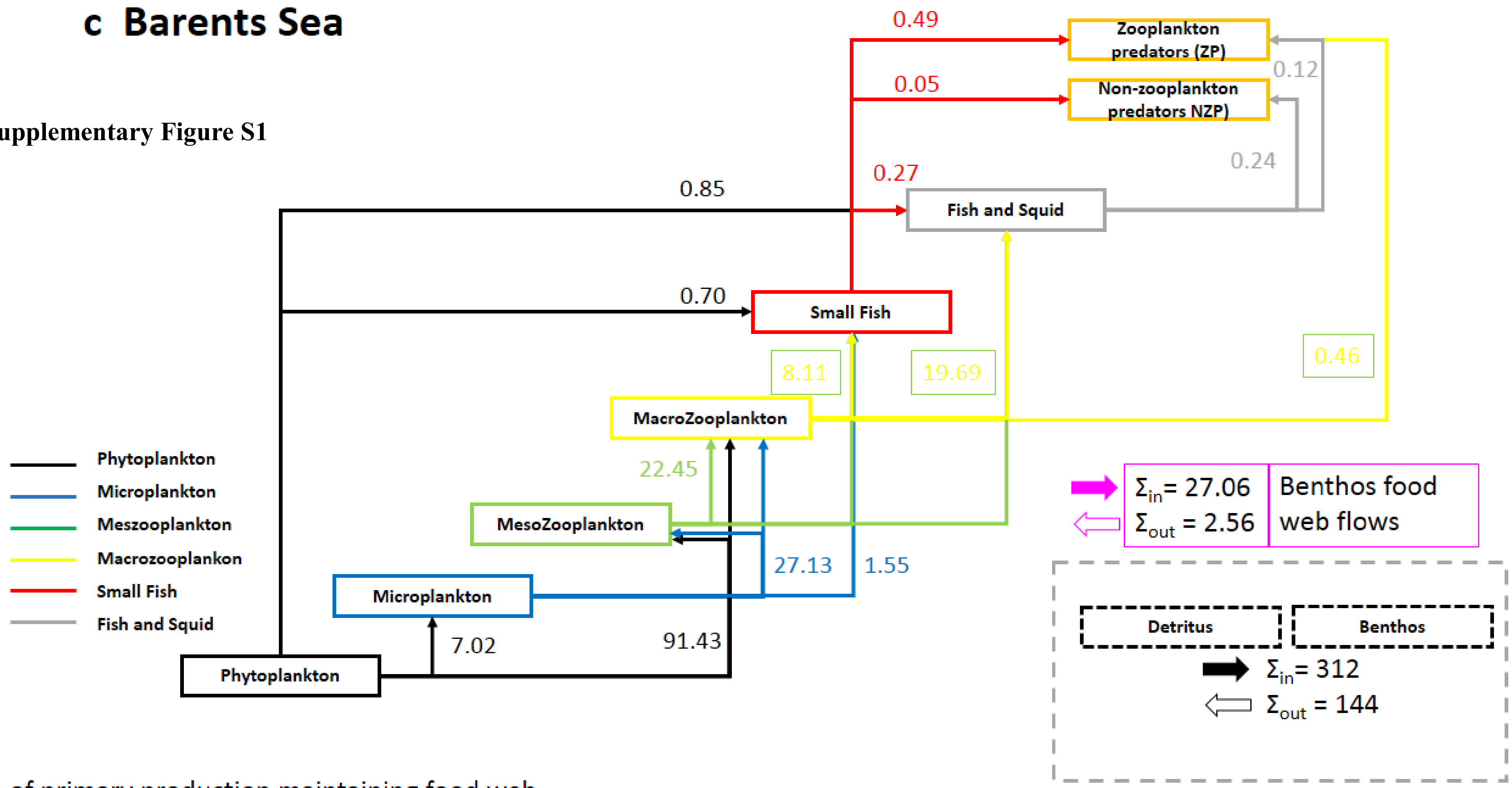

% of primary production maintaining food web

Supplementary Figure S1
